# Safety in Chemical and Biomedical Laboratories: Guidelines for the Use of Head Covers by Female Muslim Scientists

**DOI:** 10.1089/apb.2021.0015

**Published:** 2022-03-15

**Authors:** Margaret Juergensmeyer, Shakirat A. Adetunji

**Affiliations:** ^1^ Biosecurity Research Institute, Kansas State University, Manhattan, Kansas, USA.; ^2^ Department of Diagnostic Medicine/Pathobiology, Kansas State University, Manhattan, Kansas, USA.; ^3^ Center for Outcomes Research and Epidemiology, Kansas State University, Manhattan, Kansas, USA.

**Keywords:** Muslim women, personal protective equipment, PPE, respirator, head covering

## Abstract

**Introduction:**

Personal protective equipment (PPE) complements the safety measures required for working in various laboratories. The main purpose of PPE is to limit the exposure of laboratory personnel and the environment to hazardous chemicals and biological materials. Despite the wide acceptance of PPE, limited accommodation exists for customization of these items based on personal or cultural preferences.

**Discussion:**

This article describes the basic recommendations and specifications for Muslim women's head covers as a part of PPE requirements in chemical and biological laboratories.

**Conclusions:**

These guidelines will significantly help safety professionals, laboratorians, and teachers to plan accordingly and customize PPE that will not only be safe but also fit the needs of scientists of varying cultural backgrounds.

## Introduction

Personal protective equipment (PPE) is ubiquitous in laboratories. When entering a laboratory, most scientists, particularly those in the life sciences, immediately reach for a lab coat, regardless of their discipline. Although most people can easily identify with the concept of “protective” equipment, too often, the “personal” aspect of PPE is overlooked. Frequently, the word “personal” is assumed to mean “belongs to” or “the responsibility of,” instead of an arguably more important meaning, “appropriate for.” Often, it is assumed that PPE designed for one body type and need is appropriate for all individuals, and the only customization needed is a variety of sizes. Individual needs or preferences are rarely considered. For example, most researchers, given the option, exhibit a preference for a particular brand of glove, because of the way it fits their hand (Margaret Juergensmeyer, personal observation). Respirators are one of the few types of PPE where proper fit is determined by a quantitative test versus qualitative means, instead of simply requiring the user to “make do” with whatever is on-hand. Women, in particular, have noted for years that PPE of any type, from lab coats to welding masks to gloves of all sorts, if designed for men, often does not fit women properly. This is gradually changing in the manufacturing industry,^1,2^ with the emergence of PPE being designed to actually fit women, instead of merely being a smaller size and colored pink.

Body shape and size is only one facet of fitting PPE to women. Many cultures, in particular the Muslim religion, require different modesty standards for women than they do for men. Muslim women who are scientists are an ideal example of personnel caught in this type of situation. In general, Islam, a religion practiced by almost two billion people in the world,^3^ requires practicing women to implement modesty in every aspect of their lives, including clothing. One of the ways in which modesty is maintained is the mandatory use of head and body covers so that as little skin as possible is shown. Exposed skin is preferably limited to only her hands and her face from the chin with her forehead, hair, ears, neck, and shoulders being covered. In addition, for this same reason, loose-fitting clothing, which does not emphasize the body shape, is highly preferred.^4–6^ Thus, in order for Muslim women to succeed in the laboratory environment, PPE that meets their specific needs while still ensuring safety must be provided. Loose-fitting laboratory coats are reasonably easy to provide, although care must be taken to ensure that “loose fitting” does not become “dangerously dangling.” Head coverings, however, are an unfamiliar concept to many non-Muslim laboratory workers. These head covers are essential to a Muslim woman's ability to function in society in addition to the laboratory, and therefore must be considered part of her PPE in any laboratory that has clothing requirements or recommendations. Here, the authors provide a reference for the safety professional, researcher, laboratorian, and/or teacher to enable them to provide safe and effective head coverings for Muslim women in their laboratories.

## Head Coverings

Head coverings are generally formed of cloth, wrapped around the wearer's head, neck, shoulders, and chest to allow only the face from forehead to chin to be seen. Head coverings may be made of any kind of clothing material that is not transparent or translucent. They may be of any color or pattern, and many women prefer to match them to their outfits. The size is somewhat standardized, with 68″ × 34″ (173 cm × 86 cm) being common, but larger or smaller women will choose a fabric size that fits their body type. Fabric should not be overly stiff and should easily conform to the wearer's head and neck shape. Cotton or wool fabrics, or those treated with flame-resistant chemicals, should be considered, whereas nylon and other highly flammable materials should be avoided. There are companies interested in producing specific chemical-resistant and flame-resistant head coverings,^7^ but at the time this article was written, these items were not commercially available in the United States.

Reusable head coverings can be handled with the same precautions and practices that are used for lab coats. There are no restrictions on who can launder a cloth head covering. They may be laundered in-house or sent to a professional cleaner. As with most PPE, wearers would not share head coverings unless they are laundered between each use, so rapid turnaround is required. A head covering that is ripped or worn thin so that hair or skin can be seen is not usable and must be replaced.

Disposable head coverings are available from a variety of manufacturers, in a variety of styles. In general, when performing an Internet search, the search phrase “disposable head cover” is not adequate, leading mostly to shower cap style hair covers such as those worn during surgery or for food preparation. The search phrase “disposable head and neck cover” leads to a variety of appropriate types. From these, the facility can work with the women scientists to determine appropriate style, size, and cost range.

## Changing Facilities

Women who wear head coverings follow strict modesty guidelines, which are more stringent than the cultural mores of the American or Western European woman. Muslim women may not change clothes, including the head covering, in a room with people of the opposite sex who are not related to them. They are also not allowed to be naked in the presence of other women even if the other women are of their own culture/religion.^8,9^ If changing either into a different head cover or, for example, scrubs or biocontainment PPE, it is required that a private space must be provided for these women. A private room is optimal, but a women's-only restroom, even one with multiple stalls, is acceptable, as long as no other women are present in the room during changing. A lockable door is appreciated, but not required. If multiple women are expected to use a single room, time must be allowed for each of them to change individually.

## Chemical Laboratories

**Figure 1.**
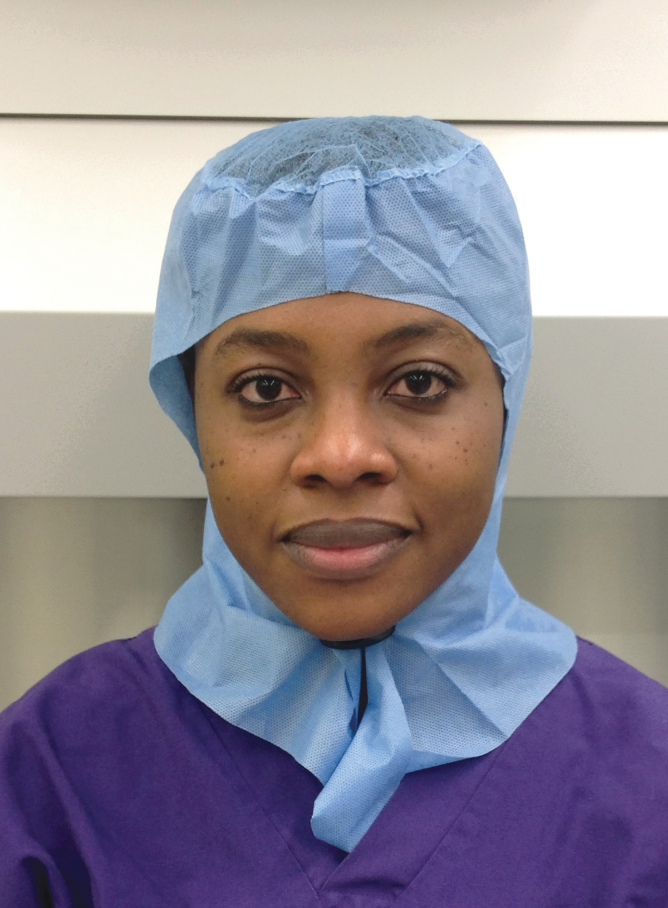
A disposable head cover, which is suitable for use by Muslim women in laboratories. Color images are available online.

In the chemical laboratory setting, one of the primary concerns is the flammability of clothing. Another concern is splashes or spills of chemicals that can stain or destroy cloth. A risk assessment of the chemicals and procedures being used should be performed before making clothing requirements/recommendations. Possible options include:

1. *Requiring that loose ends be tucked in:* The dangling ends of head coverings are usually left loose and flowing but are secured with pins so that the scarf does not come undone. Any laboratory that usually requires no dangling jewelry or loose items of clothing can easily extend this requirement to “no loose ends” of head covers. The ends can be safely and easily tucked into the collar of a lab coat or inside the wearer's shirt.
2. *Requiring flame-resistant materials:* Many head coverings are made of synthetic or synthetic blend materials. Laboratories that use burners or other flame sources should require cotton or wool head covers, to reduce the risk of fire.
3. *Requiring laboratory-specific head covers:* There is no limitation on who can purchase material for head covers, so facilities may wish to purchase head covers that they deem appropriate based on likely activities to be done in that facility. These can be one-piece, form fitting covers with no loose ends (Figure 1), or they can be of a material considered less likely to be flammable. Having laboratory-specific head covers on hand may be a very viable solution for teaching laboratories, where students may forget to wear head covers made of appropriate materials on a laboratory class day. See above for laundering and re-use requirements.
4. *Providing spare/emergency head covers:* In the event that a head cover is damaged, such as by a chemical splash or a tear, laboratories may want to consider providing emergency spare head covers. Any facility that usually maintains an extra set of clothing that can be used in the event of damage to personal clothing should also consider maintaining a spare head cover as well. Students using head covers may also be encouraged to bring their own spare.

## Biosafety Level 1/Biosafety Level 2 Laboratories

Biological laboratories, from teaching to clinical, have hazards very similar to those found in chemical laboratories, with the addition of infectious agents and/or bodily fluids. The same options discussed earlier apply, and a risk assessment performed for the individual laboratory will identify the easiest solution. In particular, attention should be paid to the risk of a splash carrying infectious material into the woman's home.

## Biocontainment Laboratories (Biosafety Level 3/Biosafety Level 4)

In biocontainment laboratories, the head cover will need to work with additional PPE such as respirators, and potential requirements for showering on entry and/or exit may also create complications. A site-specific risk assessment can again identify and resolve issues so that minor modifications are all that is needed to ensure safe use of head coverings in the laboratories, and modified procedures for individuals to shower out if required.

The authors are assuming, for the sake of this article, that no personal clothing is allowed in biocontainment laboratories. Therefore, head covers will need to be provided, either cloth that will be laundered with the facility's scrubs, or disposable. In each case, the head covers will need to be provided at the site where scrubs are acquired, and disposed of or placed for laundering at the site where scrubs are removed.

In addition, head covers of the style used in the biocontainment laboratory must be available for training and respirator fit-testing. Head covers work smoothly and easily with most PPE, but proper fit of the two in combination is essential.

**Figure 2.**
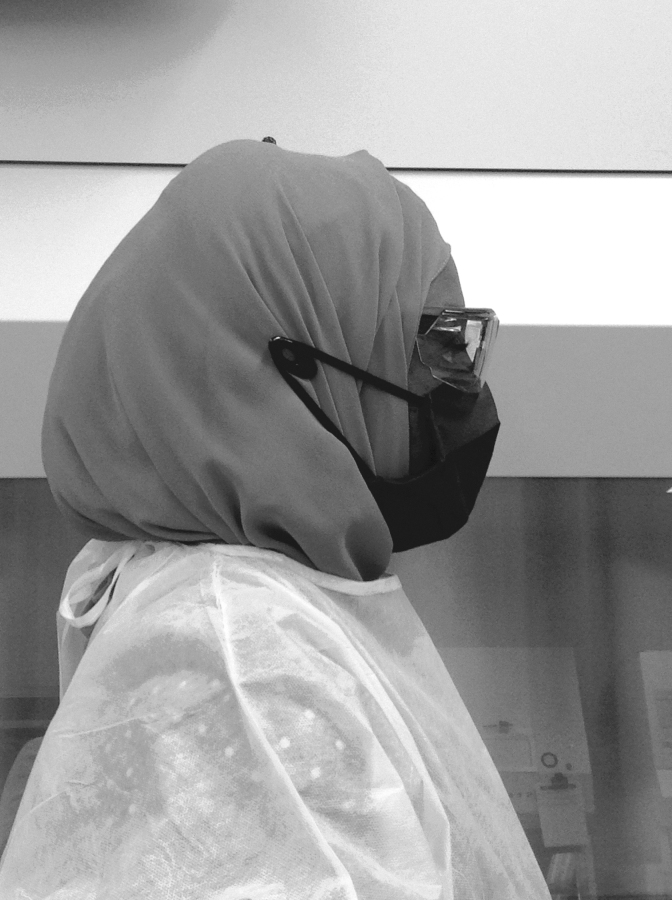
Safety glasses and a mask being worn with a cloth head cover. The earpieces of the safety glasses are worn under the head cover. Note the pins on the side of the head cover; the loops of the mask go over the pins instead of under the head cover. Color images are available online.

**Figure 3.**
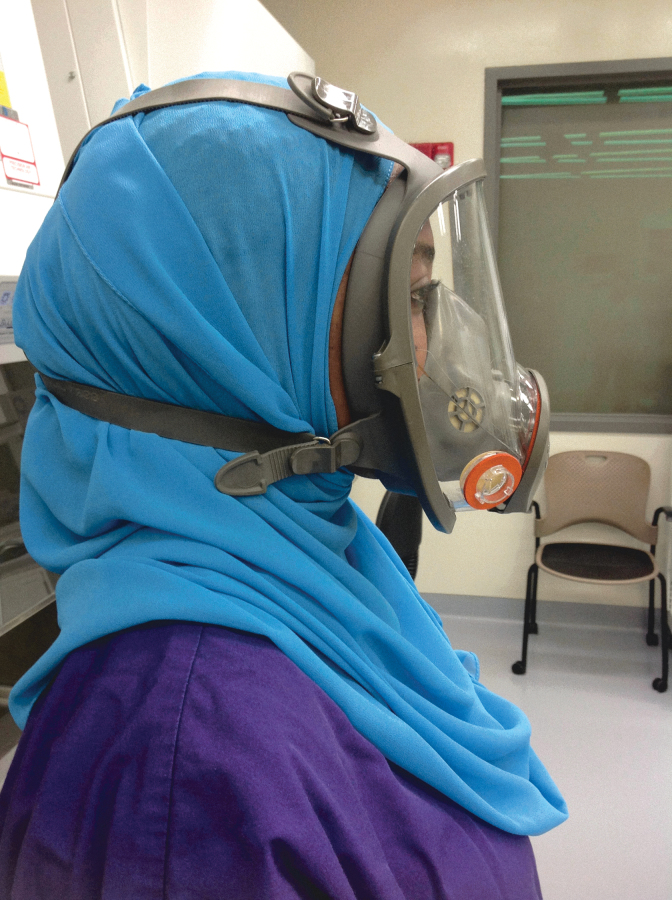
A full-face respirator being worn over a cloth head cover (cartridges not shown). The respirator straps are worn over the head cover. Respirators must be fit-tested with the type of head cover that will be used in the laboratory. Color images are available online.

**Figure 4.**
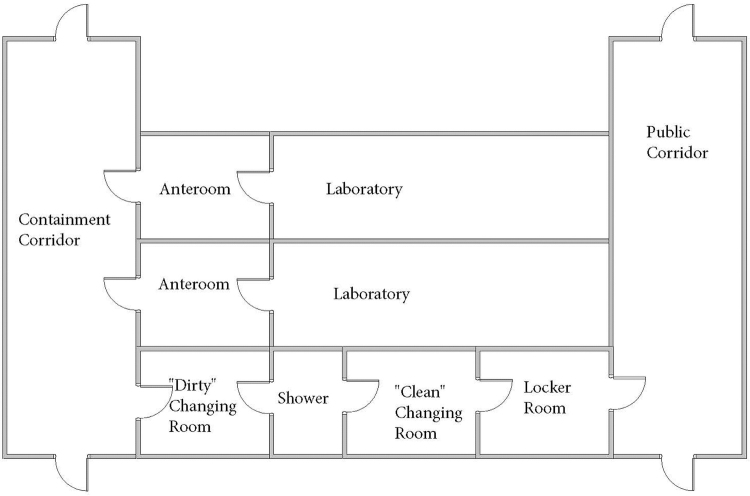
A diagram of a generic containment lab. This diagram demonstrates a number of possible areas for PPE donning/doffing. Attention should be paid to the areas in which a woman is expected to walk without a head cover (a shower can be acceptable, while a corridor is not), or to change head covers (an anteroom can be acceptable if there are no windows). In particular, the area of donning/doffing a PAPR shroud will determine whether or not a head cover must be provided for use under the PAPR shroud. PAPR, powered air-purifying respirator; PPE, personal protective equipment.

**Figure 5.**
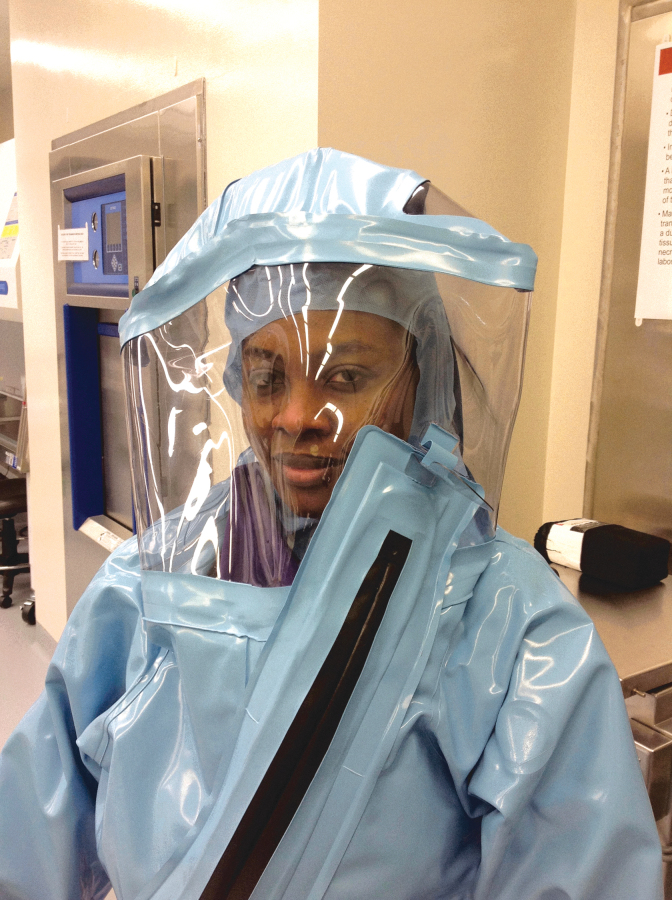
A full-body protective suit can be worn as long as a head cover is worn as well. Care needs to be taken to ensure that the head cover is not accidentally removed during donning and doffing of the suit. Color images are available online.

1. Safety glasses are worn just like normal glasses, with the earpieces of the frames going under the head cover. Goggles will have the elastic strap over the head cover, so they can be donned and doffed without affecting the head cover (Figure 2).
2. Face masks, such as surgical masks, are often designed to have loops that go behind each ear instead of completely around the head. Although it is possible to wear these loops under the head cover, donning, and particularly doffing, may pose an unacceptable risk of contamination. Pins such as those used to hold the loose ends of a head cover in place can be utilized here; when attached to the head cover just behind the ears, mask loops will hold on them (Figure 2).
3. Respirators, whether disposable or reusable, half-face or full-face, are worn with the straps over the head cover. For this reason, it is essential to provide the head cover at the time of respirator fit-testing, to ensure that the fit-test is accurate (Figure 3).
4. Powered air-purifying respirators (PAPRs) are easily used over head covers. Many of the shrouds for PAPRs can serve a dual function, both as a PAPR shroud and as a head cover. It is essential, however, if using a PAPR shroud as a head cover, to ensure that the modesty rules discussed earlier are still followed, and the wearer is not expected to walk down a “clean” hallway or be in any other non-private location without a head cover. Figure 4 shows a generic containment facility, with a locker room, a shower for exiting, and multiple laboratories. Disposable head covers and/or PAPR shrouds can be staged in a wide variety of ways, depending on how the facility is run. For example, if the normal routine of the laboratory is to change into scrubs in the “clean” changing room, walk down the containment corridor, and then enter an anteroom to put on PPE for a specific laboratory, the wearer of a head cover will need a separate head cover provided in the “clean” changing room. This allows the PAPR to be put on and taken off in the anteroom. Alternatively, if the PAPR is put on in the “dirty” changing room and then used at all times in containment, moving from lab to lab, a head cover will only be needed if the PAPR shroud is loose-fitting and does not adequately cover the hair, head, and neck. It can be very beneficial to sit down with a person requiring a head cover and a map of the facility, and to discuss where both routine and emergency head covers could be staged.
5. Full-body protective suits such as those worn in BSL-4 suit laboratories are easily used with the same disposable hair cover used in laboratory work. Care should be taken during fitting of the suit to ensure that the head area will not accidentally catch on the hair cover (Figure 5).

## Emergency Response

In an emergency, the head cover may be accidentally or purposefully removed. Preservation of life and health take precedence over modesty, as the Islamic religion is not intended as a hardship for anyone^10,11^; however, there are some modifications/customizations that can be offered to a woman wearing a head cover that will greatly improve her emotional comfort.

Treat any event in which a head cover is removed just like an event in which a woman's shirt is removed. If the removal is accidental and involves no injury (e.g., head cover snagged on equipment), offer a private place to replace it, such as an office or even a blanket held up to shield her from onlookers. All personnel, women included, should be strongly encouraged to look away while the woman corrects her clothing.

Emergency exiting of a laboratory usually requires removal of PPE, particularly contaminated PPE. If a head cover must be removed due to contamination or to treat an injury, provide as much privacy as possible. If the woman must be moved through public areas, such as on a stretcher, even a towel or blanket covering her hair, neck, and shoulders is appreciated. If possible, female medical staff are appreciated; however, life safety is more important than modesty, so do not hesitate to involve male medical staff.

## Conclusion

Students, scientists, and laboratory officials must work together to ensure the protection of all personnel, equipment, and the environment. Appropriate PPE that accommodates the varying needs of diverse students and scientific staff must be provided by the institutions. As described earlier, provision of culturally appropriate PPE for Muslim women requires only a small number of resources, in both time and money. Although this article focuses solely on the needs of Muslim women, other cultures with specific clothing or modesty requirements can also benefit from having a safety professional sit down with one or more members of the culture, and respectfully discuss cultural requirements, safety requirements, and to mutually determine the best resources to achieve both goals simultaneously. These resources, when provided, will be repaid many times over by ensuring that a vital and growing segment of the laboratory population can flourish.
